# Legal Medicine

**Published:** 1895-01

**Authors:** 


LEGAL MEDICINE, Hamilton's System of, Assisted by Thirty Medical and Legal Collaborators, is announced as now ready. Complete in two volumes, $5.50 each.

